# Plasma-based COVID-19 treatments in low-and middle-income countries and the risk of transfusion-transmitted infections

**DOI:** 10.1038/s41541-020-00256-6

**Published:** 2020-11-03

**Authors:** Jay Epstein, W. Martin Smid, Silvano Wendel, Daniel Somuah, Thierry Burnouf

**Affiliations:** 1 US Food and Drug Administration, Silver Spring, MD USA; 2 Sanquin Consulting Services, Amsterdam, Academic Institute IDTM, Groningen, The Netherlands; 3 Blood Bank, Hospital Sírio-Libanês, São Paulo, Brasil; 4 Anglogold Ashanti Health Foundation Hospital, Obuasi, Ghana; 5 Graduate Institute of Biomedical Materials and Tissue Engineering & International PhD Program in Biomedical Engineering, College of Biomedical Engineering, Taipei Medical University, Taipei, Taiwan

**Keywords:** Diseases, Risk factors, Medical research

## Abstract

Ethical principles should prevail in the collection, testing and use of COVID-19 convalescent plasma (CCP) for human research in low- and middle- income countries. To appropriately guarantee safety, only blood establishments that comply with recognized quality standards should collect CCP.

In their commentary recently published in *npj Vaccines*^1^, Ferreira and Mostajo-Radji call attention to the risk for transmission of blood-borne infections, especially HIV, that may be associated with administration of COVID-19 convalescent plasma (CCP) in low- and middle-income countries (LMIC). They note that transfusion-transmitted HIV aggravates community spread of HIV. Absence of universal HIV testing in LMIC, inability to provide appropriate antibody and PCR-based testing, and pressures for paid plasma donations are identified as factors contributing to this risk. At the same time, deficiencies in availability of COVID-19 testing, including tests for neutralizing antibodies, limit the potential benefits for use of CCP in these settings.

Members of the Working Party on Global Blood Safety (WP GBS) of the International Society of Blood Transfusion have recognized the importance to assure “Non-reactivity of blood samples for transfusion transmitted infections including HIV, HBV, HCV, syphilis (for whole blood) and locally transmitted infections using approved serological and/or nucleic acid tests, consistent with local requirements for collection of blood components for transfusion”^2^. Nevertheless, the 2015 WHO Global Database for Blood Safety documented less than 100% donation testing for one or more major disease agents in some LMIC countries^3^. The survey indicated that “in some countries not all quality-assured testing procedures were followed; more particularly, donated blood was reported as 100% tested by 99.8% of respondents in high-income countries; by 99.9% of respondents in upper middle-income countries; by 83% of respondents in lower middle-income countries; and by 76% of respondents in low-income countries. Furthermore, 28 countries reported stock-outs of screening assays (one in the South-East Asia Region, two in the Eastern Mediterranean Region, three in the European Region, five in the Region of the Americas, six in the Western Pacific Region and 11 countries in the African Region). The use of rapid tests for all or part of the blood donations was reported in 25 of 141 countries (three in the Eastern Mediterranean Region, four in South-East Asia Region, nine countries in the African Region, and nine in the Western Pacific Region). Most of these 25 countries are low-income (eight countries) and lower middle-income (15 countries). Widespread use of less sensitive rapid diagnostic tests in many low- and middle-income countries contributes to risk of transfusion-transmissible infections, as does use of suboptimal testing strategies (for example, with regard to the choice of tests and testing algorithms)”^3^.

Donation testing to prevent transmission of blood-borne infections including HIV is among the most basic standards in modern blood banking. Furthermore, ethical principles imply that safety and efficacy should be maximized for collection and use of CCP as an experimental product^4^. For these reasons, the WP GBS has argued that preparation and use of CCP should take place in nationally coordinated initiatives under which recognized quality standards are met by blood establishments^5^. Where feasible, infectious safety of CCP can be further optimized by processing the collection with a validated method of pathogen inactivation^6^.

WHO recently published an “Action Framework to Advance Universal Access to Safe, Effective and Quality-Assured Blood Products 2020–2023” that provides strategic direction to global efforts to overcome barriers to the safety and availability of blood and blood products that persist in many countries^3^. The six strategic objectives are “(1) an appropriately structured, well-coordinated and sustainably resourced national blood system; (2) an appropriate national framework of regulatory controls, national standards and quality assessment programmes; (3) functioning and efficiently managed blood services; (4) effective implementation of patient blood management to optimize clinical practice of transfusion; (5) effective surveillance, haemovigilance and pharmacovigilance, supported by comprehensive and accurate data collection systems; and (6) partnerships, collaboration and information exchange to achieve key priorities and jointly address challenges and emerging threats at global, regional and national levels”^3^. Strengthening national blood systems including blood regulation is a prerequisite for improving national responses to crises affecting blood product safety and availability, including provision of convalescent plasma against COVID-19 and future measures that will be needed to address other emerging infections that are potentially transmissible by blood or may impact blood service operations^7^.
